# Comparison of the effect of different infusion rates of sufentanil on surgical stress index during cranial pinning in children under general anaesthesia: a randomized controlled study

**DOI:** 10.1186/s12871-017-0448-6

**Published:** 2017-12-08

**Authors:** In-Kyung Song, Sang-Hwan Ji, Eun-Hee Kim, Ji-Hyun Lee, Jin-Tae Kim, Hee-Soo Kim

**Affiliations:** 1 Department of Anaesthesiology and Pain Medicine, Asan Medical Center, Seoul, Republic of Korea; 2 Department of Anaesthesiology and Pain Medicine, College of Medicine, Seoul National University, Seoul, Republic of Korea

**Keywords:** Children, Opioid, Surgical stress index

## Abstract

**Background:**

Surgical stress index (SSI) is an established indicator for intraoperative nociception. Opioids are used to block stimulus of cranial pinning in neurosurgery. We investigated the effect of different infusion rates of sufentanil on SSI during cranial pinning in children under general anaesthesia.

**Methods:**

Forty-nine children (2–12 years of age) underwent neurosurgery with pinning. The children were randomized into three groups based on the rate of sufentanil infusion: 0.2, 0.5, and 0.8 μg·kg^−1^·hr.^−1^. Continuous sufentanil infusion began following neuromuscular blockade administration, at a rate determined by each patient’s assigned treatment group. Following preparation for surgery, cranial pinning was performed. Systolic, diastolic, and mean blood pressures, along with heart rate and photoplethysmographic data, were continuously recorded from 1 min prior to cranial pinning through 5 min after cranial pinning, in 1-min intervals. SSI was calculated following the completion of surgery. Differences in measured outcomes over time among the three groups were evaluated using a generalized estimation equation. Differences in pinning outcomes in the same group were evaluated with Freidman test.

**Results:**

We found no statistical differences in long-term SSI that were associated with different infusion rates of sufentanil during cranial pinning. Blood pressures in all groups increased for 2 min after cranial pinning, and then decreased; we found no statistical difference in long-term blood pressure values among the groups. Heart rate increased after pinning in the group that received a low-dose infusion of sufentanil.

**Conclusions:**

Since SSI was intended to measure the blunting effects of sufentanil towards the noxious stimulus of cranial pinning, our results suggest that SSI might not be sufficiently sensitive to monitor the nociceptive response in children.

**Trial registration:**

(KCT0000978, Jan-07, 2014).

## Background

Pain is a term that describes an emotional and personal experience. However, patients cannot express a conscious sense of surgical pain during general anaesthesia. Nonetheless, patients can respond to surgical stimuli; this response appears to be blunted by the administration of anaesthesia. Importantly, insufficient management of the nociceptive response can affect postoperative outcomes [1–4]. Therefore, maintenance of the balance between nociception and antinociception is important during anaesthesia. Traditionally, autonomic responses, such as tachycardia, hypertension, or sweating, have been used to assess nociception during general anaesthesia. However, the reliability of these responses may vary because of potential confounders [5, 6].

Recently, a variety of methods have been suggested to monitor nociception during anaesthesia: pupillometry [7], surgical pleth index (SPI) [8], surgical stress index (SSI) [9], skin conductance [10], analgesia/nociception index [11], cardiovascular depth of analgesia index [12], wavelet transform cardiorespiratory coherence [13], photoplethysmogram amplitude (PPGA) [14], and nociception level index [15]. However, as most of these measurements require specific monitoring devices, they may be impossible to implement without the aid of specific devices that are not available in every clinical setting. Among the measurements listed above, SSI based on photoplethysmogram (PPG) may best facilitate monitoring of nociception during anaesthesia because all patients are monitored by PPG during anaesthesia using standard devices that are present in a wide range of clinical settings.

Pinning for head fixation (also known as cranial pinning) during neurosurgery is a very short and strong stimulus; the responses to cranial pinning under insufficient analgesia might include hypertension, tachycardia, increased intracranial pressure, or disturbance of cerebral perfusion [16]. Additionally, sufentanil is an opioid that is commonly used to blunt the noxious stimulus during neurosurgical anaesthesia; its infusion rate is typically adjusted according to blood pressure (BP) or heart rate (HR). However, BP and HR may not be appropriate reference measurements because they might be affected by stimuli other than the balance between nociception and anti-nociception, such as volume state or use of vasopressors.

In this study, we investigated the effect of different infusion rates of sufentanil on SSI, which is a known method for intraoperative nociceptive monitoring, during cranial pinning in children under general anaesthesia.

## Methods

### Ethics, consent and permissions

This study was approved by the institutional review board of Seoul National University Hospital (H-1310-044-526, Seoul, Korea) and registered at cris.nih.go.kr (KCT0000978, Jan-07, 2014).

After obtaining informed consent from parents or guardians of children who were scheduled for elective neurosurgery under general anaesthesia, we enrolled 51 children (2–12 years of age) who had a physical status of 2 or 3, per guidelines from the American Society of Anesthesiologists. All surgeries included cranial pinning prior to the neurosurgical procedure. Exclusion criteria included known peripheral vascular disease, cardiovascular disease, respiratory disease, increased intracranial pressure or an allergy to opioids.

**Fig. 1:**
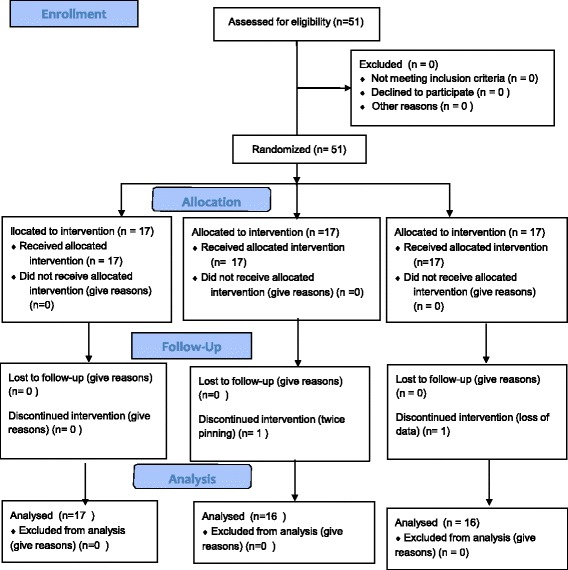
CONSORT diagram showing flow of patients through the various stages of the study

Patients were randomly assigned to three groups prior to surgery (https://www.randomizer.org/) (Fig. 1: CONSORT diagram): 0.2 μg·kg^−1^·hr.^−1^ of sufentanil administration, group L; 0.5 μg·kg^−1^·hr.^−1^ of sufentanil administration, group M; 0.8 μg·kg^−1^·hr.^−1^ of sufentanil administration, group H. An equal number of patients were assigned to each group.

Each patient was fasted per preoperative fasting guidelines and arrived at the operating theatre without premedication. The patients were appropriately hydrated with intravenous Ringer’s lactate solution, according to Holliday-Segar guideline [17]; this was confirmed by acceptable initial vital signs, which were compared with ward values. Each patient was transported to the operating room and monitored by electrocardiograph (ECG), non-invasive blood pressure, pulse oximeter (SpO_2_) on the finger, end-tidal carbon dioxide (E_T_CO_2_) with patient monitor (Solar 8000; GE Medical, Milwaukee, WI, USA), and Bispectral Index™ (BIS; Covidien, Mansfield, MA, USA). Anaesthesia was induced with atropine (0.02 mg·kg^−1^, 0.5 mg maximal dose) and sodium thiopental (5 mg·kg^−1^) in patients who were <3 years old; in patients who were 3–12 years old, anaesthesia was induced with propofol (2–2.5 mg·kg^−1^). Patients were ventilated with 8% sevoflurane in 100% oxygen (vol/vol). Tracheal intubation was performed after full relaxation via administration of 0.6 mg/kg of rocuronium. Subsequently, sufentanil was administered through an infusion port directly connected to the patient, per the assigned treatment group. General anaesthesia was maintained with sevoflurane or desflurane in air with 35% oxygen, and ventilation was adjusted to maintain 35–40 mmHg of E_T_CO_2_. The concentration of inhalation agents was adjusted to achieve 1–1.5 minimal alveolar concentration of inhalational agents per the patient’s vital signs and BIS values (40–60), as well as at the discretion of the attending anaesthesiologist.

Cranial pinning was performed by the attending surgeon after local infiltration with lidocaine and at least 1 h following the start of sufentanil administration. After the pinning procedure, no stimuli were applied to each patient for a period of 5 min. Conventional surgical procedures were performed and patients were transferred to the post-anaesthetic care unit or intensive care unit after the completion of surgical procedures.

### Data collection

All patients’ data, including ECG and PPG recordings, were collected and transferred from the patient’s monitor to the personal computer using analogue-to-digital converter (DA 149, DATAQ Instruments, Akron, OH, USA) at 1000 Hz. The data collection period was from 1 min prior to cranial pinning through 5 min after cranial pinning; during this 5-min period, no additional stimulation was provided to each patient. Additionally, BP and HR were recorded in 1-min intervals during the same period of ECG data collection. The SSI is a dimensionless number between 0 (low stress) and 100 (high stress) that is calculated from the ECG and the PPG with 8-s data averaging that are performed after data is obtained. The precise algorithm was described in a previous report [16].

The calculation of SSI is below:

$$ \mathrm{SSI}=100-\left(0.{33}^{\ast }{\mathrm{PBI}}_{\mathrm{norm}}+0.{67}^{\ast }{\mathrm{PPGA}}_{\mathrm{norm}}\right), $$

where PBI_norm_ represents the normalized pulse beat interval (PBI) and PPGA_norm_ represents the normalized PPGA from the PPG.

Our primary outcome was change in SSI during cranial pinning among the three treatment groups in 1-min intervals over time; the secondary outcomes were changes in systolic BP (SBP), mean BP (MBP), diastolic BP (DBP), and HR over time. Additionally, changes in BP, HR, and SSI over time, within each group, were sub-analysed.

### Sample size estimation and statistics

We based our sample size calculations on a previous study performed on adult patients [14], as there was no similar study in children, and used G*Power© software (version 3.1, Franz Faul, Universitaet Kiel, Germany). Differences in mean-to-peak ratios of PPG were assumed to be 0.02 and the standard deviation was assumed to be 0.02, according to the previous study. Our thresholds were set at a power level of 0.8 and a significance level of 0.017 (for 3 groups); at least 15 patients were needed in each group, and a total of 51 patients was required because of an expected attrition rate of 10% in each group.

We tested the normality of our data distribution using the Shapiro–Wilk test. Differences in demographic data, primary outcomes, and secondary outcomes among three groups over time were evaluated using generalized estimation equations, as data were not normally distributed. Additionally, the pinning-associated change in parameters was evaluated using the Friedman test with post-hoc analysis. Data are presented as median [Inter-quartile range] or numbers, as appropriate. A value of *p* < 0.05 was considered to indicate statistical significance. All statistical analyses were performed using SPSS software (SPSS 21.0, IBM Inc., Chicago, Illinois, USA).

## Results

In total, 51 patients were enrolled, and 49 patients (group L; 17, group M; 16, group H; 16) completed the study. One patient was excluded because of an excess of two cranial pinning attempts, and the other patient was removed from the study because of loss of patient data. Demographic data indicated no differences among the three groups (Table 1).

**Table 1:** Demographic characteristics

	Group L (*N* = 17)	Group M (*N* = 16)	Group H (*N* = 16)
Age	7[5–10]	6[4–9.6]	6.8[3.6–10.2]
Weight	20.0[18.1–25]	21.3[16.3–29.7]	22.9[17.4–31.1]
Height	114.2[102.4–127.4]	114.8[110.8–132.4]	116.9[112.8–140.0]
Inhalational agent (Sevoflurane/ desflurane)	10/7	10/6	9/7

Values are median [interquartile range:IQR]

The calculated SSI during cranial pinning of head fixation did not indicate differences among the groups over time. Although the baseline value of SSI in group L (57.8[50.9–63.4; IQR]) was slightly higher than SSI in other groups (52.6[50.4–55.2; IQR] in group M, 53.4[50.3–59.4; IQR] in group H), there were no significant differences across all groups. Further, SSI values decreased over time but there were also no differences among the groups (Table 2).

**Table 2:** Changes of surgical stress index during pinning

	low	mid	high
Baseline	57.8 [50.9–63.4]	52.6 [50.4–55.2]	53.4 [50.3–59.4]
1	54.7 [48.9–58.1]	51.8 [45.1–55.3]	52.8[43.7–58.2]
2	53..0 [49.5–53.1]	49.2 [45.90–51.9]	50.7 [43.1–56.0]
3	51.2 [48.9–57.3]	51.4 [46.9–56.4]	52.1 [41.3–57.5]
4	49.3 [44.50–59.0]	48.5 [43.2–52.8]	51.2 [47.5–57.1]
5	52.6 [47.1–56.7]	47.5 [38.2–54.7]	48.1 [43.3–61.9]

Baseline SBP, MBP and DBP were similar among the three groups. The values of SBP, MBP, and DBP significantly increased at both 1 and 2 min following cranial pinning in all three groups, with the exception of DBP in group M at 2 min after pinning; after this initial period, all BP values subsequently decreased (Table 3). Importantly, we observed no differences in BP among the three groups at all measured time points.

**Table 3:** Changes of blood pressure during pinning

Time(min)		Group L	Group M	Group H
baseline	SBP	109.6[105.2–130.1]	104.8 [98.5–112.6]	99.2[91.6–117.6]
	MBP	83.3[73.9–93.6]	74.9[69.0–82.2]	70.1[62.2–80.8]
	DBP	66.7[56.9–74.9]	59.5[55.1–64.4]	59.3[48.0–65.2]
1	SBP	132.6[113.1–143.4]**	118.0[106.2–136.2]*	105.4[99.3–130.0]**
	MBP	101.0[85.6–109.3]**	92.7[79.6–1056.0]**	83.2[74.2–99.7]**
	DBP	82.8[69.1–93.5]**	73.5[64.9–88.3]*	68.2[59.5–83.8]**
2	SBP	134.3[111.7–140.4]*	114.7[104.1–130.6]	107.3[99.7–133.1]**
	MBP	93.1[79.7–111.2]**	88.5[74.6–98.2]*	78.7[70.1–101.4]*
	DBP	76.3[64.0–90.0]*	69.9[60.5–80.8]	63.4[58.6–83.1]*
3	SBP	126.9[111.6–138.1]	112.8[99.0–124.0]	108.8[98.9–123.8]
	MBP	90.2[77.1–99.3]	84.8[71.3–95.5]	80.1[68.9–90.0]
	DBP	73.9[60.2–78.0]	67.5[58.0–76.2]	65.4[56.1–72.9]
4	SBP	117.7[110.4–131.1]	108.2[96.1–118.1]	105.9[96.6–111.7]
	MBP	86.9[75.7–96.5]	79.5[69.1–89.0]	78.1[67.6–80.7]
	DBP	69.5[57.9–77.3]	62.8[54.2–70.9]	59.91[55.0–69.7]
5	SBP	112.5[108.0–125.0]	105.4[94.2–113.8]	103.8[94.8–106.9]
	MBP	83.0[73.6–92.1]	76.1[67.9–84.7]	71.6[66.9–79.2]
	DBP	64.4[56.8–72.1]	58.3[53.1–68.0]	57.0[54.3–68.1]

Values are median [interquartile range:IQR]

*P < 0.05 compared to baseline values

**P < 0.01 compared to baseline values

In addition, baseline HR did not demonstrate differences among the three groups. Further, HR only increased in group L at 1 and 2 min after cranial pinning, after which it returned to baseline values. In groups M and H, there was weak statistical evidence for a trend towards an increase in HR. We observed differences in HR among the three groups over time; these also demonstrated weak statistical evidence (Table 4). Although the HR decreased after cranial pinning, there was no significant bradycardia that required clinical management.

**Table 4:** Changes of heart rate during pinning

	low	mid	high
Baseline	105.3[89.3–134.6]	103.0[89.4–146.8]	100.9[86.3–126.2]
1	124.2[94.1–145.6]**	116.6[92.9–166.4]	108.2[92.1–136.2]
2	115.8[93.3–145.5]*	114.7[90.7–161.7]	101.1[89.4–128.0]
3	103.1[92.0–135.2]	118.0[89.8–153.2]	103.9[80.0–124.2]
4	97.2[92.0–130.9]	113.3[87.6–149.9]	103.8[77.8–122.6]
5	94.4[89.3–130.0]	111.0[87.3–146.5]	102.3[76.7–121.3]

Values are median [interquartile range:IQR]

*P < 0.05 compared to baseline values

**P < 0.01 compared to baseline values

## Discussion

In this study, SSI changes did not vary with the infusion rate of sufentanil used for prevention of the noxious cranial pinning stimulus in children during general anaesthesia.

Currently, nociceptive response monitoring remains a challenge during general anaesthesia. In the past, SSI, which was derived from the photoplethysmographic waveform amplitude and heart beat-to-beat intervals, was used as a surrogate marker of analgesia [18].

SSI is a composite measurement that may be used to monitor the patient’s hemodynamic responses to surgical stimuli and analgesic medications during general anaesthesia. It reflects the patient’s responses, which result from increased sympathetic activity as a reaction to nociceptive stimuli. A previous study showed that multiple stress indicators (SSI, PPGA, HR, BP, response entropy and state entropy) succeeded in detecting the nociceptive stimulus caused by intubation and surgery in anaesthetized children [19]. These findings indicated a clinical use for SSI during general anaesthesia in children, suggesting that SSI might be useful as an indicator of nociceptive stimulus. Therefore, we hypothesized that, if high dose of opioid could block the nociceptive stimulus, a patient’s SSI might remain stable in the presence of a high opioid dose; in contrast, a low dose of opioid would not prevent changes in SSI. Here, we compared different infusion rates of opioid (sufentanil) with SSI values, along with traditional parameters such as BP and HR, to measure nociceptive response. We used cranial pinning as the nociceptive stimulus during general anaesthesia. However, we found no differences in SSI values in the presence of differing infusion rates of sufentanil, in contrast to the previous study; however, other traditional parameters might vary over time during cranial pinning.

In our study, BP changed with the cranial pinning stimulus, demonstrating an increase at 1 and 2 min after cranial pinning in all groups; subsequently, BP decreased. Importantly, this finding was consistent with a previous study [20]. However, SSI did not follow this trend, thereby demonstrating that SSI measurements and traditional clinical findings were quite disparate. This can be explained in multiple ways. First, the algorithm to calculate SSI is based on a ‘normalized’ PPGA wave (67% included in the final index) and pulse beat interval (PBI, 33%); however, this algorithm is proprietary and does not include BP [21], which may explain the lack of differences seen in SSI. Another possible explanation is that there is an uncertainty in SSI due to the nociceptive response in children. Lastly, we checked BP and averaged SSI, both in 1 min intervals. Therefore, we might have missed changes in SSI by averaging the SSI values, which were calculated every 8 s, even though the noxious stimulus of cranial pinning was very short. Interestingly, HR increased in low-dose infusion of sufentanil, unlike in the other two groups, despite there being no differences among the three groups.

Interestingly, HR only increased in the low-dose group (0.2 μg·kg^−1^·hr.^−1^) immediately after pinning, and then decreased to baseline. Across all three groups, BP changed after pinning, while HR increased only in the low-dose group. A previous study showed that decreases in HR were sustained and were achieved more rapidly than changes in BP that were achieved with the same dose of sufentanil [22]. Therefore, sufentanil seems to affect HR more profoundly than BP. In addition, another study of sternotomy patients reported that HR did not change in a group that was treated with sufentanil, compared with a group that was treated with both remifentanil and sufentanil; however, SBP and MBP did change in both groups [23]. These findings demonstrate different responses in HR and BP after administration of the same dose of opioid. This might be similar to our observations of no changes in HR upon noxious stimulus, but clear changes in BP with high dose of opioid. These differences might be explained by factors that influence BP and HR. Notably, cardiac output, total peripheral resistance, arterial stiffness and clinical states are factors that influence BP. In contrast, HR is influenced by hormones, electrolytes, chemoreceptors and baroreceptors. Therefore, opioids might differently influence each component that affects BP or HR.

There are several studies that have used SPI as the nociceptive monitor. As the algorithm to calculate SPI is essentially the same as that used to calculate SSI, findings from studies that use SPI might be applicable to the results in our present study. A previous study showed that SPI had moderate correlation with stress hormones during anaesthesia, thereby indicating that SPI could predict adrenocorticotropin values with high sensitivity and specificity [24]. Interestingly, the SPI group (remifentanil administration adjusted based on SPI values) and the control group (remifentanil administration adjusted based on traditional signs and symptoms, such as heart rate and blood pressure) did not show differences in SPI values between baseline and the intubation event; further, SPI decreased at maximal surgical stimulus. Although the report did not provide the concentration of remifentanil of the two groups, SPI values were similar between baseline and the intubation event, suggesting that the remifentanil could prevent a nociceptive response to intubation. In addition, the previous report indicated that intubation stimulus was the most powerful pain according to SPI measurements, because the maximal surgical stimulus exhibited a lower SPI value. In our study, SSI was similar between baseline and post-cranial pinning measurements, so we infer that the stimulus of cranial pinning is similar to an intubation event. However, changes in blood pressure during our study were not consistent with the previous report. On the contrary, blood pressure was increased 1 min after cranial pinning and immediately decreased, suggesting that this clinical assessment might be more sensitive than SSI. This discrepancy might be explained by the subjects recruited in that study (adults vs. children) and the type of noxious stimulus. Other reports have shown that SPI is influenced by several factors, such as posture and anaesthetic technique, and that changes in SPI and heart rate were not correlated each other [25]. Therefore, SPI and clinical findings, such as heart rate, are not always matched; this is consistent with our study.

Before we obtained clinical data, sufentanil was administered at the same concentration for at least 1 h. As we did not check the sufentanil concentration, we estimated each patient’s sufentanil concentration using Guay’s paediatric sufentanil model [26]; sufentanil concentrations required nearly 45 min to reach steady-state between plasma and effect-site concentrations.

The limitations of our study include our failure to measure the blood concentration of sufentanil. Although the duration of sufentanil administration was sufficient to achieve steady-state and we estimated sufentanil concentration for each patient, we did not check the blood concentration of sufentanil. Therefore, the three groups could not be distinguished according to the real concentration of sufentanil, but could be differentiated on the basis of the initial dose of sufentanil given to patients. The other limitation is the different force of cranial pinning, according to the attending surgeon. Although the force was not identical in all patients, SSI or other clinical response did not differ across the three groups, and thus, the pinning force did not affect the results. Another limitation is the administration of atropine; although it might cause significant HR variability or changes in SSI, we administered atropine to all patients to reduce bias in the data. Lastly, we did not check the other markers of stress response, such as plasma catecholamine levels, to confirm stress in our patient population.

## Conclusions

In conclusion, SSI changes during cranial pinning under general anaesthesia were not different due to different doses of sufentanil administration.

## Abbreviations

BIS: Bispectral Index™
BP: Blood pressure
DBP: Diastolic blood pressure
ECG: Electrocardiograph
E_T_CO_2_: End-tidal carbon dioxide
HR: Heart rate
MBP: Mean blood pressure
PBI: Pulse beat interval
PPG: Photoplethysmogram
PPGA: Photoplethysmogram amplitude
SBP: Systolic blood pressure
SSI: Surgical stress index
